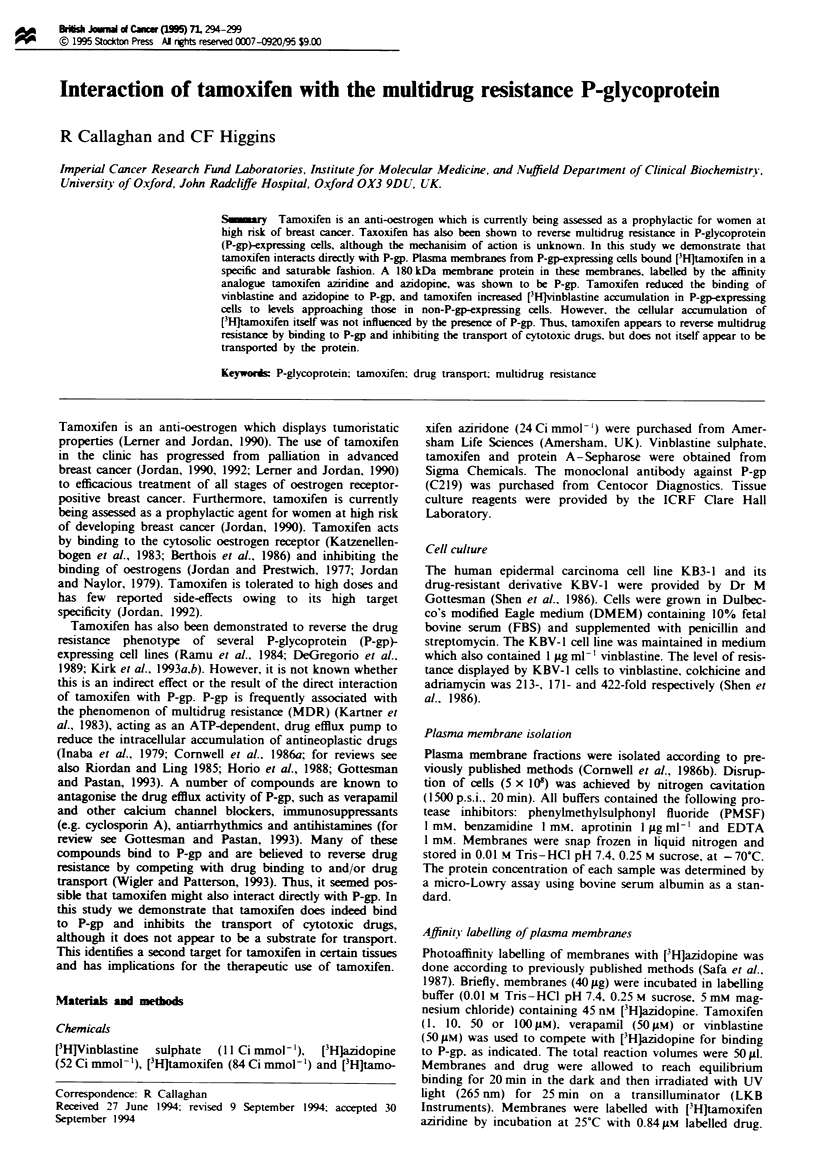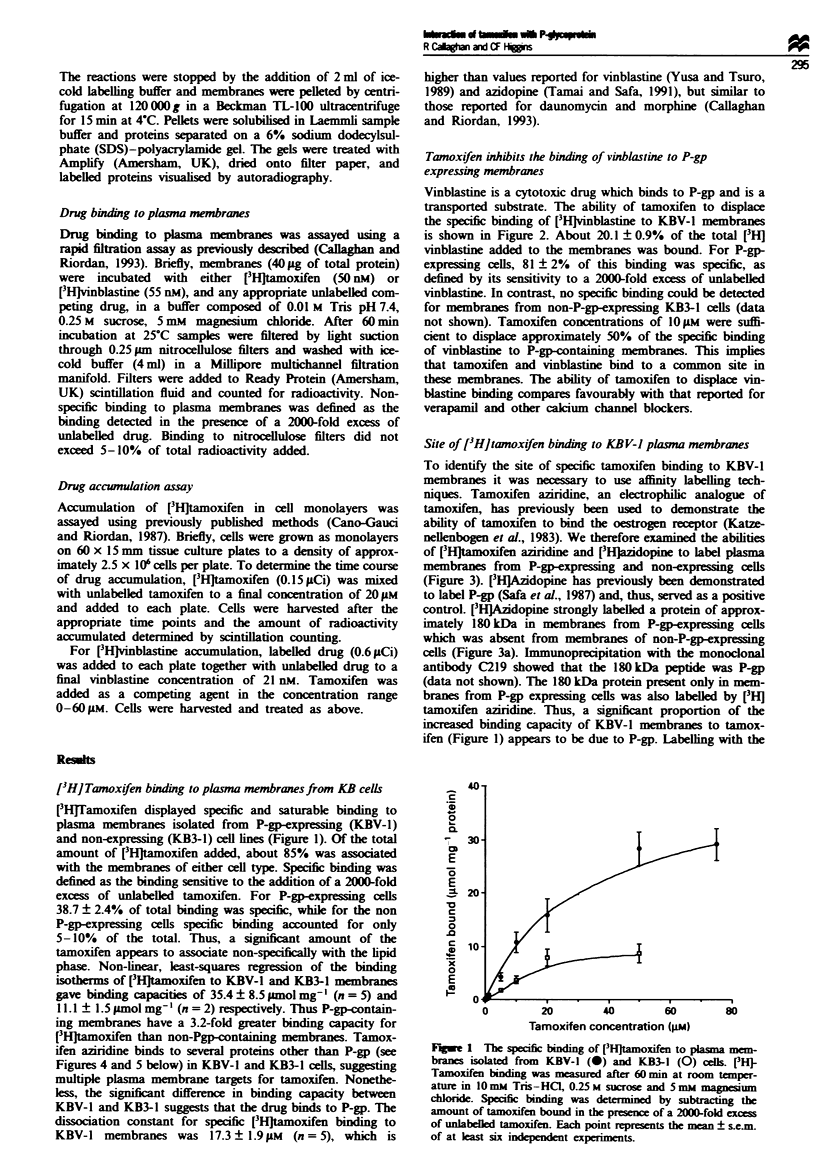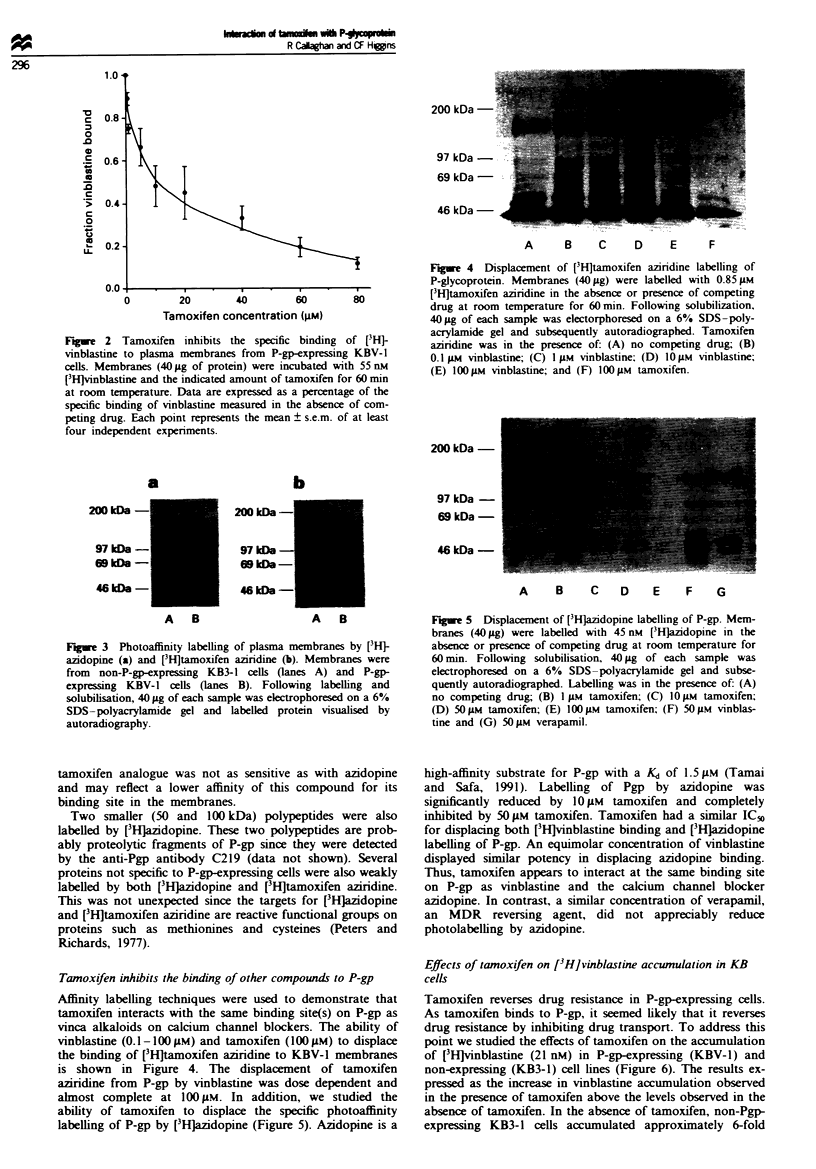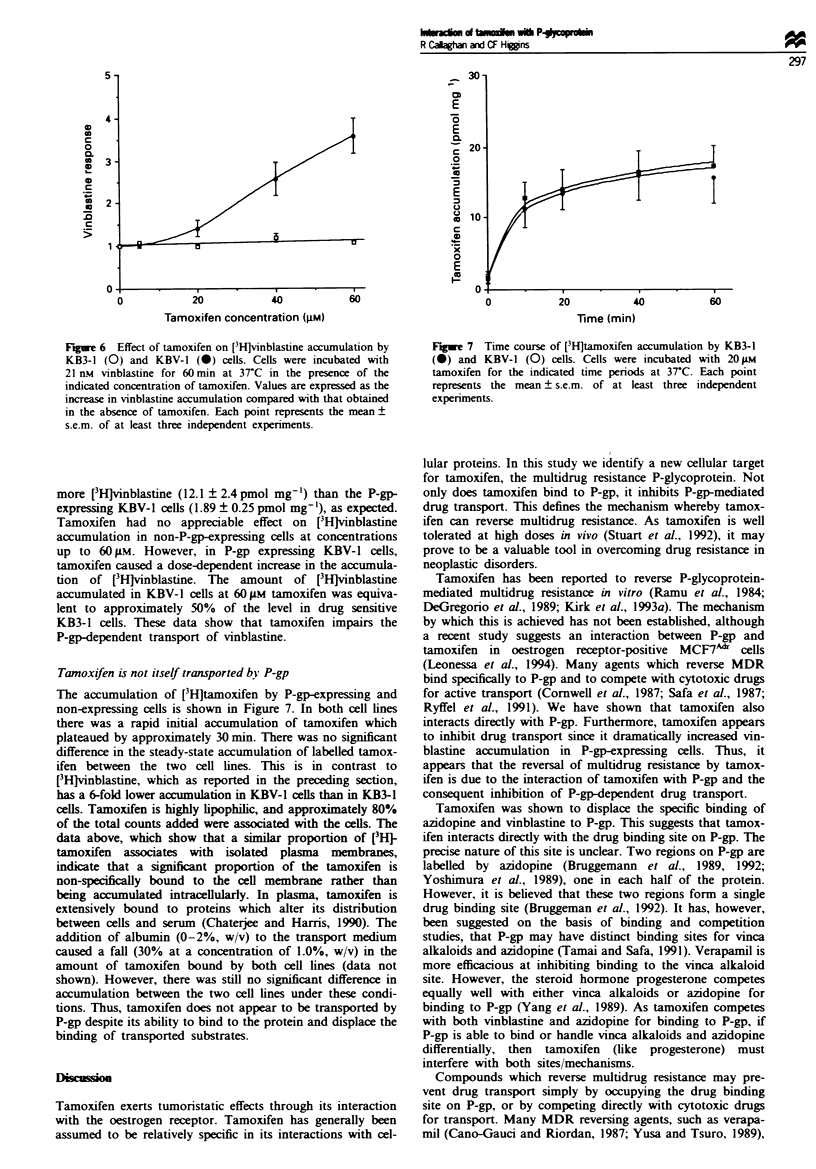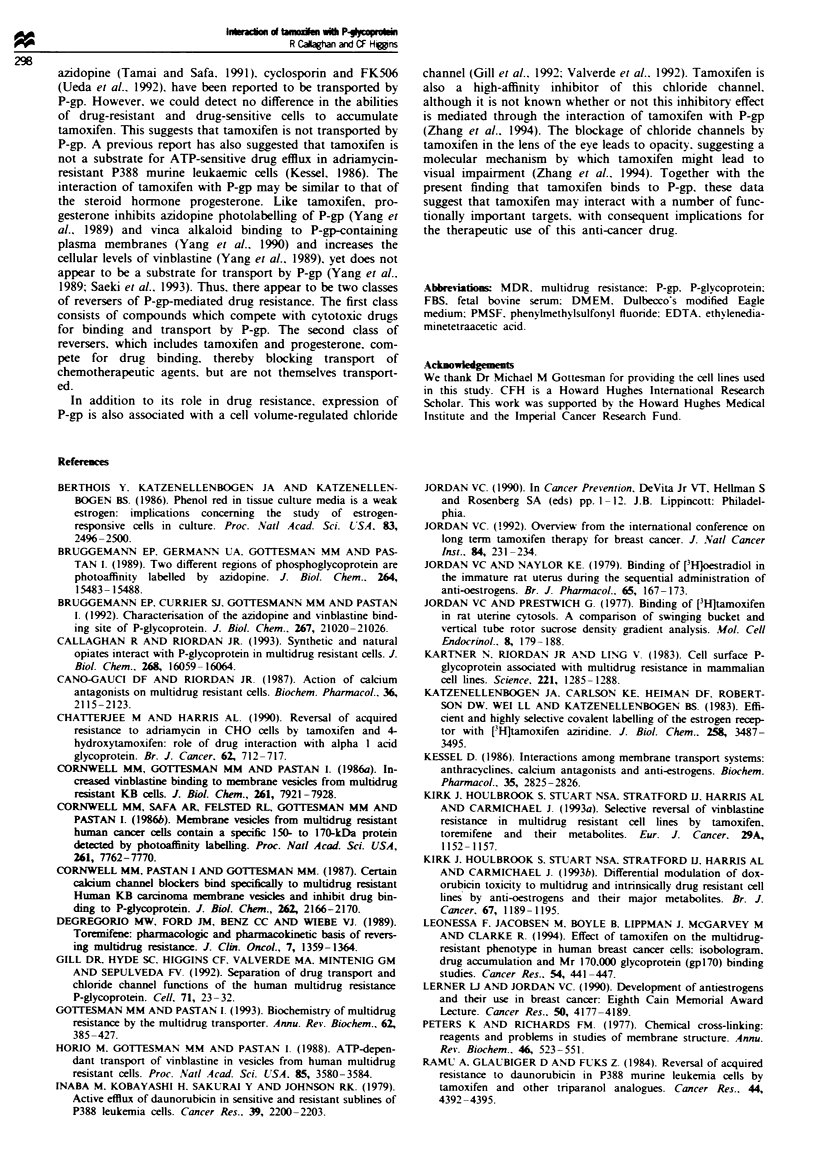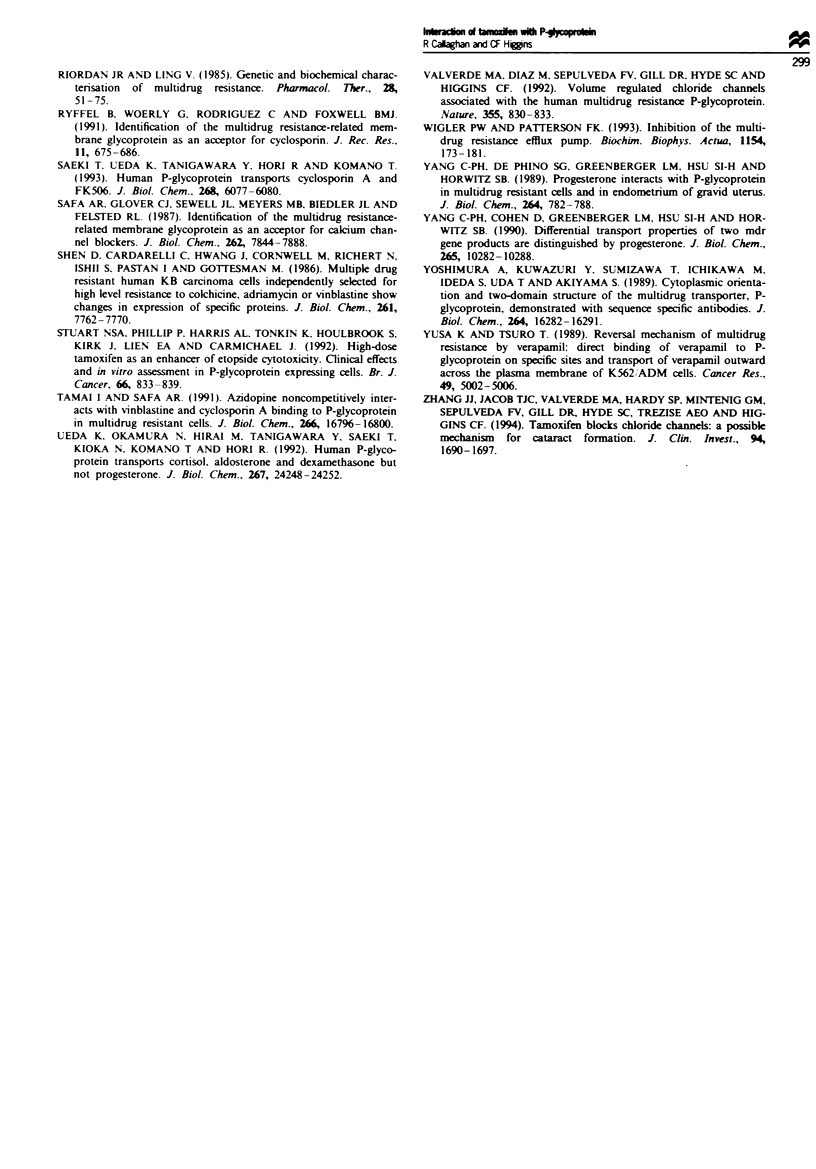# Interaction of tamoxifen with the multidrug resistance P-glycoprotein.

**DOI:** 10.1038/bjc.1995.59

**Published:** 1995-02

**Authors:** R. Callaghan, C. F. Higgins

**Affiliations:** Imperial Cancer Research Fund Laboratories, Institute for Molecular Medicine, Oxford, UK.

## Abstract

**Images:**


© 1995 Stockton Press All rights reserved 0007-0920/95 $9.00

### Interaction of tamoxifen with the multidrug resistance P-glycoprotein

R Callaghan and CF Higgins

*Imperial Cancer Research Fund Laboratories, Institute for Molecular Medicine, and Nuffield Department of Clinical Biochemistry, University of Oxford, John Radcliffe Hospital, Oxford OX3 9DU, UK.*

**Summary** Tamoxifen is an anti-oestrogen which is currently being assessed as a prophylactic for women at high risk of breast cancer. Taxoxifen has also been shown to reverse multidrug resistance in P-glycoprotein (P-gp)-expressing cells, although the mechanisim of action is unknown. In this study we demonstrate that tamoxifen interacts directly with P-gp. Plasma membranes from P-gp-expressing cells bound [$^3$H]tamoxifen in a specific and saturable fashion. A 180 kDa membrane protein in these membranes, labelled by the affinity analogue tamoxifen aziridine and azidopine, was shown to be P-gp. Tamoxifen reduced the binding of vinblastine and azidopine to P-gp, and tamoxifen increased [$^3$H]vinblastine accumulation in P-gp-expressing cells to levels approaching those in non-P-gp-expressing cells. However, the cellular accumulation of [$^3$H]tamoxifen itself was not influenced by the presence of P-gp. Thus, tamoxifen appears to reverse multidrug resistance by binding to P-gp and inhibiting the transport of cytotoxic drugs, but does not itself appear to be transported by the protein.

**Keywords:** P-glycoprotein; tamoxifen; drug transport; multidrug resistance

Tamoxifen is an anti-oestrogen which displays tumoristatic properties (Lerner and Jordan, 1990). The use of tamoxifen in the clinic has progressed from palliation in advanced breast cancer (Jordan, 1990, 1992; Lerner and Jordan, 1990) to efficacious treatment of all stages of oestrogen receptor-positive breast cancer. Furthermore, tamoxifen is currently being assessed as a prophylactic agent for women at high risk of developing breast cancer (Jordan, 1990). Tamoxifen acts by binding to the cytosolic oestrogen receptor (Katzenellenbogen *et al.*, 1983; Berthois *et al.*, 1986) and inhibiting the binding of oestrogens (Jordan and Prestwich, 1977; Jordan and Naylor, 1979). Tamoxifen is tolerated to high doses and has few reported side-effects owing to its high target specificity (Jordan, 1992).

Tamoxifen has also been demonstrated to reverse the drug resistance phenotype of several P-glycoprotein (P-gp)-expressing cell lines (Ramu *et al.*, 1984; DeGregorio *et al.*, 1989; Kirk *et al.*, 1993*a*,*b*). However, it is not known whether this is an indirect effect or the result of the direct interaction of tamoxifen with P-gp. P-gp is frequently associated with the phenomenon of multidrug resistance (MDR) (Kartner *et al.*, 1983), acting as an ATP-dependent, drug efflux pump to reduce the intracellular accumulation of antineoplastic drugs (Inaba *et al.*, 1979; Cornwell *et al.*, 1986*a*; for reviews see also Riordan and Ling 1985; Horio *et al.*, 1988; Gottesman and Pastan, 1993). A number of compounds are known to antagonise the drug efflux activity of P-gp, such as verapamil and other calcium channel blockers, immunosuppressants (e.g. cyclosporin A), antiarrhythmics and antihistamines (for review see Gottesman and Pastan, 1993). Many of these compounds bind to P-gp and are believed to reverse drug resistance by competing with drug binding to and/or drug transport (Wigler and Patterson, 1993). Thus, it seemed possible that tamoxifen might also interact directly with P-gp. In this study we demonstrate that tamoxifen does indeed bind to P-gp and inhibits the transport of cytotoxic drugs, although it does not appear to be a substrate for transport. This identifies a second target for tamoxifen in certain tissues and has implications for the therapeutic use of tamoxifen.

### Materials and methods

### Chemicals

[$^3$H]Vinblastine sulphate (11 Ci mmol$^{-1}$), [$^3$H]azidopine (52 Ci mmol$^{-1}$), [$^3$H]tamoxifen (84 Ci mmol$^{-1}$) and [$^3$H]tamoxifen aziridone (24 Ci mmol$^{-1}$) were purchased from Amersham Life Sciences (Amersham, UK). Vinblastine sulphate, tamoxifen and protein A-Sepharose were obtained from Sigma Chemicals. The monoclonal antibody against P-gp (C219) was purchased from Centocor Diagnostics. Tissue culture reagents were provided by the ICRF Clare Hall Laboratory.

### Cell culture

The human epidermal carcinoma cell line KB3-1 and its drug-resistant derivative KBV-1 were provided by Dr M Gottesman (Shen *et al.*, 1986). Cells were grown in Dulbecco's modified Eagle medium (DMEM) containing 10% fetal bovine serum (FBS) and supplemented with penicillin and streptomycin. The KBV-1 cell line was maintained in medium which also contained 1 $\mu$g ml$^{-1}$ vinblastine. The level of resistance displayed by KBV-1 cells to vinblastine, colchicine and adriamycin was 213-, 171- and 422-fold respectively (Shen *et al.*, 1986).

### Plasma membrane isolation

Plasma membrane fractions were isolated according to previously published methods (Cornwell *et al.*, 1986b). Disruption of cells ($5 \times 10^8$) was achieved by nitrogen cavitation (1500 p.s.i., 20 min). All buffers contained the following protease inhibitors: phenylmethylsulphonyl fluoride (PMSF) 1 mM, benzamidine 1 mM, aprotinin 1 $\mu$g ml$^{-1}$ and EDTA 1 mM. Membranes were snap frozen in liquid nitrogen and stored in 0.01 M Tris-HCl pH 7.4, 0.25 M sucrose, at $-70$°C. The protein concentration of each sample was determined by a micro-Lowry assay using bovine serum albumin as a standard.

### Affinity labelling of plasma membranes

Photoaffinity labelling of membranes with [$^3$H]azidopine was done according to previously published methods (Safa *et al.*, 1987). Briefly, membranes (40 $\mu$g) were incubated in labelling buffer (0.01 M Tris-HCl pH 7.4, 0.25 M sucrose, 5 mM magnesium chloride) containing 45 nM [$^3$H]azidopine. Tamoxifen (1, 10, 50 or 100 $\mu$M), verapamil (50 $\mu$M) or vinblastine (50 $\mu$M) was used to compete with [$^3$H]azidopine for binding to P-gp, as indicated. The total reaction volumes were 50 $\mu$l. Membranes and drug were allowed to reach equilibrium binding for 20 min in the dark and then irradiated with UV light (265 nm) for 25 min on a transilluminator (LKB Instruments). Membranes were labelled with [$^3$H]tamoxifen aziridine by incubation at 25°C with 0.84 $\mu$M labelled drug.

Correspondence: R Callaghan
Received 27 June 1994; revised 9 September 1994; accepted 30 September 1994

The reactions were stopped by the addition of 2 ml of ice-cold labelling buffer and membranes were pelleted by centrifugation at 120 000 g in a Beckman TL-100 ultracentrifuge for 15 min at 4°C. Pellets were solubilised in Laemmli sample buffer and proteins separated on a 6% sodium dodecylsulphate (SDS)-polyacrylamide gel. The gels were treated with Amplify (Amersham, UK), dried onto filter paper, and labelled proteins visualised by autoradiography.

*Drug binding to plasma membranes*

Drug binding to plasma membranes was assayed using a rapid filtration assay as previously described (Callaghan and Riordan, 1993). Briefly, membranes (40 μg of total protein) were incubated with either [$^3$H]tamoxifen (50 nM) or [$^3$H]vinblastine (55 nM), and any appropriate unlabelled competing drug, in a buffer composed of 0.01 M Tris pH 7.4, 0.25 M sucrose, 5 mM magnesium chloride. After 60 min incubation at 25°C samples were filtered by light suction through 0.25 μm nitrocellulose filters and washed with ice-cold buffer (4 ml) in a Millipore multichannel filtration manifold. Filters were added to Ready Protein (Amersham, UK) scintillation fluid and counted for radioactivity. Non-specific binding to plasma membranes was defined as the binding detected in the presence of a 2000-fold excess of unlabelled drug. Binding to nitrocellulose filters did not exceed 5-10% of total radioactivity added.

*Drug accumulation assay*

Accumulation of [$^3$H]tamoxifen in cell monolayers was assayed using previously published methods (Cano-Gauci and Riordan, 1987). Briefly, cells were grown as monolayers on 60 × 15 mm tissue culture plates to a density of approximately 2.5 × $10^6$ cells per plate. To determine the time course of drug accumulation, [$^3$H]tamoxifen (0.15 μCi) was mixed with unlabelled tamoxifen to a final concentration of 20 μM and added to each plate. Cells were harvested after the appropriate time points and the amount of radioactivity accumulated determined by scintillation counting.

For [$^3$H]vinblastine accumulation, labelled drug (0.6 μCi) was added to each plate together with unlabelled drug to a final vinblastine concentration of 21 nM. Tamoxifen was added as a competing agent in the concentration range 0-60 μM. Cells were harvested and treated as above.

### Results

*[$^3$H]Tamoxifen binding to plasma membranes from KB cells*

[$^3$H]Tamoxifen displayed specific and saturable binding to plasma membranes isolated from P-gp-expressing (KBV-1) and non-expressing (KB3-1) cell lines (Figure 1). Of the total amount of [$^3$H]tamoxifen added, about 85% was associated with the membranes of either cell type. Specific binding was defined as the binding sensitive to the addition of a 2000-fold excess of unlabelled tamoxifen. For P-gp-expressing cells 38.7 ± 2.4% of total binding was specific, while for the non P-gp-expressing cells specific binding accounted for only 5-10% of the total. Thus, a significant amount of the tamoxifen appears to associate non-specifically with the lipid phase. Non-linear, least-squares regression of the binding isotherms of [$^3$H]tamoxifen to KBV-1 and KB3-1 membranes gave binding capacities of 35.4 ± 8.5 μmol mg$^{-1}$ ($n = 5$) and 11.1 ± 1.5 μmol mg$^{-1}$ ($n = 2$) respectively. Thus P-gp-containing membranes have a 3.2-fold greater binding capacity for [$^3$H]tamoxifen than non-Pgp-containing membranes. Tamoxifen aziridine binds to several proteins other than P-gp (see Figures 4 and 5 below) in KBV-1 and KB3-1 cells, suggesting multiple plasma membrane targets for tamoxifen. Nonetheless, the significant difference in binding capacity between KBV-1 and KB3-1 suggests that the drug binds to P-gp. The dissociation constant for specific [$^3$H]tamoxifen binding to KBV-1 membranes was 17.3 ± 1.9 μM ($n = 5$), which is higher than values reported for vinblastine (Yusa and Tsuro, 1989) and azidopine (Tamai and Safa, 1991), but similar to those reported for daunomycin and morphine (Callaghan and Riordan, 1993).

*Tamoxifen inhibits the binding of vinblastine to P-gp expressing membranes*

Vinblastine is a cytotoxic drug which binds to P-gp and is a transported substrate. The ability of tamoxifen to displace the specific binding of [$^3$H]vinblastine to KBV-1 membranes is shown in Figure 2. About 20.1 ± 0.9% of the total [$^3$H] vinblastine added to the membranes was bound. For P-gp-expressing cells, 81 ± 2% of this binding was specific, as defined by its sensitivity to a 2000-fold excess of unlabelled vinblastine. In contrast, no specific binding could be detected for membranes from non-P-gp-expressing KB3-1 cells (data not shown). Tamoxifen concentrations of 10 μM were sufficient to displace approximately 50% of the specific binding of vinblastine to P-gp-containing membranes. This implies that tamoxifen and vinblastine bind to a common site in these membranes. The ability of tamoxifen to displace vinblastine binding compares favourably with that reported for verapamil and other calcium channel blockers.

*Site of [$^3$H]tamoxifen binding to KBV-1 plasma membranes*

To identify the site of specific tamoxifen binding to KBV-1 membranes it was necessary to use affinity labelling techniques. Tamoxifen aziridine, an electrophilic analogue of tamoxifen, has previously been used to demonstrate the ability of tamoxifen to bind the oestrogen receptor (Katzenellenbogen *et al.*, 1983). We therefore examined the abilities of [$^3$H]tamoxifen aziridine and [$^3$H]azidopine to label plasma membranes from P-gp-expressing and non-expressing cells (Figure 3). [$^3$H]Azidopine has previously been demonstrated to label P-gp (Safa *et al.*, 1987) and, thus, served as a positive control. [$^3$H]Azidopine strongly labelled a protein of approximately 180 kDa in membranes from P-gp-expressing cells which was absent from membranes of non-P-gp-expressing cells (Figure 3a). Immunoprecipitation with the monoclonal antibody C219 showed that the 180 kDa peptide was P-gp (data not shown). The 180 kDa protein present only in membranes from P-gp expressing cells was also labelled by [$^3$H] tamoxifen aziridine. Thus, a significant proportion of the increased binding capacity of KBV-1 membranes to tamoxifen (Figure 1) appears to be due to P-gp. Labelling with the

**Figure 1** The specific binding of [$^3$H]tamoxifen to plasma membranes isolated from KBV-1 (●) and KB3-1 (○) cells. [$^3$H]-Tamoxifen binding was measured after 60 min at room temperature in 10 mM Tris-HCl, 0.25 M sucrose and 5 mM magnesium chloride. Specific binding was determined by subtracting the amount of tamoxifen bound in the presence of a 2000-fold excess of unlabelled tamoxifen. Each point represents the mean ± s.e.m. of at least six independent experiments.

**Figure 2** Tamoxifen inhibits the specific binding of [$^3$H]-vinblastine to plasma membranes from P-gp-expressing KBV-1 cells. Membranes (40 µg of protein) were incubated with 55 nM [$^3$H]vinblastine and the indicated amount of tamoxifen for 60 min at room temperature. Data are expressed as a percentage of the specific binding of vinblastine measured in the absence of competing drug. Each point represents the mean ± s.e.m. of at least four independent experiments.

**Figure 3** Photoaffinity labelling of plasma membranes by [$^3$H]-azidopine (**a**) and [$^3$H]tamoxifen aziridine (**b**). Membranes were from non-P-gp-expressing KB3-1 cells (lanes A) and P-gp-expressing KBV-1 cells (lanes B). Following labelling and solubilisation, 40 µg of each sample was electrophoresed on a 6% SDS-polyacrylamide gel and labelled protein visualised by autoradiography.

**Figure 4** Displacement of [$^3$H]tamoxifen aziridine labelling of P-glycoprotein. Membranes (40 µg) were labelled with 0.85 µM [$^3$H]tamoxifen aziridine in the absence or presence of competing drug at room temperature for 60 min. Following solubilization, 40 µg of each sample was electorphoresed on a 6% SDS-polyacrylamide gel and subsequently autoradiographed. Tamoxifen aziridine was in the presence of: (A) no competing drug; (B) 0.1 µM vinblastine; (C) 1 µM vinblastine; (D) 10 µM vinblastine; (E) 100 µM vinblastine; and (F) 100 µM tamoxifen.

**Figure 5** Displacement of [$^3$H]azidopine labelling of P-gp. Membranes (40 µg) were labelled with 45 nM [$^3$H]azidopine in the absence or presence of competing drug at room temperature for 60 min. Following solubilisation, 40 µg of each sample was electrophoresed on a 6% SDS-polyacrylamide gel and subsequently autoradiographed. Labelling was in the presence of: (A) no competing drug; (B) 1 µM tamoxifen; (C) 10 µM tamoxifen; (D) 50 µM tamoxifen; (E) 100 µM tamoxifen; (F) 50 µM vinblastine and (G) 50 µM verapamil.

tamoxifen analogue was not as sensitive as with azidopine and may reflect a lower affinity of this compound for its binding site in the membranes.

Two smaller (50 and 100 kDa) polypeptides were also labelled by [$^3$H]azidopine. These two polypeptides are probably proteolytic fragments of P-gp since they were detected by the anti-Pgp antibody C219 (data not shown). Several proteins not specific to P-gp-expressing cells were also weakly labelled by both [$^3$H]azidopine and [$^3$H]tamoxifen aziridine. This was not unexpected since the targets for [$^3$H]azidopine and [$^3$H]tamoxifen aziridine are reactive functional groups on proteins such as methionines and cysteines (Peters and Richards, 1977).

### *Tamoxifen inhibits the binding of other compounds to P-gp*

Affinity labelling techniques were used to demonstrate that tamoxifen interacts with the same binding site(s) on P-gp as vinca alkaloids on calcium channel blockers. The ability of vinblastine (0.1–100 µM) and tamoxifen (100 µM) to displace the binding of [$^3$H]tamoxifen aziridine to KBV-1 membranes is shown in Figure 4. The displacement of tamoxifen aziridine from P-gp by vinblastine was dose dependent and almost complete at 100 µM. In addition, we studied the ability of tamoxifen to displace the specific photoaffinity labelling of P-gp by [$^3$H]azidopine (Figure 5). Azidopine is a high-affinity substrate for P-gp with a $K_d$ of 1.5 µM (Tamai and Safa, 1991). Labelling of Pgp by azidopine was significantly reduced by 10 µM tamoxifen and completely inhibited by 50 µM tamoxifen. Tamoxifen had a similar $IC_{50}$ for displacing both [$^3$H]vinblastine binding and [$^3$H]azidopine labelling of P-gp. An equimolar concentration of vinblastine displayed similar potency in displacing azidopine binding. Thus, tamoxifen appears to interact at the same binding site on P-gp as vinblastine and the calcium channel blocker azidopine. In contrast, a similar concentration of verapamil, an MDR reversing agent, did not appreciably reduce photolabelling by azidopine.

### *Effects of tamoxifen on [$^3$H]vinblastine accumulation in KB cells*

Tamoxifen reverses drug resistance in P-gp-expressing cells. As tamoxifen binds to P-gp, it seemed likely that it reverses drug resistance by inhibiting drug transport. To address this point we studied the effects of tamoxifen on the accumulation of [$^3$H]vinblastine (21 nM) in P-gp-expressing (KBV-1) and non-expressing (KB3-1) cell lines (Figure 6). The results expressed as the increase in vinblastine accumulation observed in the presence of tamoxifen above the levels observed in the absence of tamoxifen. In the absence of tamoxifen, non-Pgp-expressing KB3-1 cells accumulated approximately 6-fold

**Figure 6** Effect of tamoxifen on [$^3$H]vinblastine accumulation by KB3-1 (○) and KBV-1 (●) cells. Cells were incubated with 21 nM vinblastine for 60 min at 37°C in the presence of the indicated concentration of tamoxifen. Values are expressed as the increase in vinblastine accumulation compared with that obtained in the absence of tamoxifen. Each point represents the mean ± s.e.m. of at least three independent experiments.

**Figure 7** Time course of [$^3$H]tamoxifen accumulation by KB3-1 (●) and KBV-1 (○) cells. Cells were incubated with 20 μM tamoxifen for the indicated time periods at 37°C. Each point represents the mean ± s.e.m. of at least three independent experiments.

more [$^3$H]vinblastine (12.1 ± 2.4 pmol mg$^{-1}$) than the P-gp-expressing KBV-1 cells (1.89 ± 0.25 pmol mg$^{-1}$), as expected. Tamoxifen had no appreciable effect on [$^3$H]vinblastine accumulation in non-P-gp-expressing cells at concentrations up to 60 μM. However, in P-gp expressing KBV-1 cells, tamoxifen caused a dose-dependent increase in the accumulation of [$^3$H]vinblastine. The amount of [$^3$H]vinblastine accumulated in KBV-1 cells at 60 μM tamoxifen was equivalent to approximately 50% of the level in drug sensitive KB3-1 cells. These data show that tamoxifen impairs the P-gp-dependent transport of vinblastine.

*Tamoxifen is not itself transported by P-gp*

The accumulation of [$^3$H]tamoxifen by P-gp-expressing and non-expressing cells is shown in Figure 7. In both cell lines there was a rapid initial accumulation of tamoxifen which plateaued by approximately 30 min. There was no significant difference in the steady-state accumulation of labelled tamoxifen between the two cell lines. This is in contrast to [$^3$H]vinblastine, which as reported in the preceding section, has a 6-fold lower accumulation in KBV-1 cells than in KB3-1 cells. Tamoxifen is highly lipophilic, and approximately 80% of the total counts added were associated with the cells. The data above, which show that a similar proportion of [$^3$H]-tamoxifen associates with isolated plasma membranes, indicate that a significant proportion of the tamoxifen is non-specifically bound to the cell membrane rather than being accumulated intracellularly. In plasma, tamoxifen is extensively bound to proteins which alter its distribution between cells and serum (Chaterjee and Harris, 1990). The addition of albumin (0–2%, w/v) to the transport medium caused a fall (30% at a concentration of 1.0%, w/v) in the amount of tamoxifen bound by both cell lines (data not shown). However, there was still no significant difference in accumulation between the two cell lines under these conditions. Thus, tamoxifen does not appear to be transported by P-gp despite its ability to bind to the protein and displace the binding of transported substrates.

### Discussion

Tamoxifen exerts tumoristatic effects through its interaction with the oestrogen receptor. Tamoxifen has generally been assumed to be relatively specific in its interactions with cellular proteins. In this study we identify a new cellular target for tamoxifen, the multidrug resistance P-glycoprotein. Not only does tamoxifen bind to P-gp, it inhibits P-gp-mediated drug transport. This defines the mechanism whereby tamoxifen can reverse multidrug resistance. As tamoxifen is well tolerated at high doses *in vivo* (Stuart *et al.*, 1992), it may prove to be a valuable tool in overcoming drug resistance in neoplastic disorders.

Tamoxifen has been reported to reverse P-glycoprotein-mediated multidrug resistance *in vitro* (Ramu *et al.*, 1984; DeGregorio *et al.*, 1989; Kirk *et al.*, 1993*a*). The mechanism by which this is achieved has not been established, although a recent study suggests an interaction between P-gp and tamoxifen in oestrogen receptor-positive MCF7$^{Adr}$ cells (Leonessa *et al.*, 1994). Many agents which reverse MDR bind specifically to P-gp and to compete with cytotoxic drugs for active transport (Cornwell *et al.*, 1987; Safa *et al.*, 1987; Ryffel *et al.*, 1991). We have shown that tamoxifen also interacts directly with P-gp. Furthermore, tamoxifen appears to inhibit drug transport since it dramatically increased vinblastine accumulation in P-gp-expressing cells. Thus, it appears that the reversal of multidrug resistance by tamoxifen is due to the interaction of tamoxifen with P-gp and the consequent inhibition of P-gp-dependent drug transport.

Tamoxifen was shown to displace the specific binding of azidopine and vinblastine to P-gp. This suggests that tamoxifen interacts directly with the drug binding site on P-gp. The precise nature of this site is unclear. Two regions on P-gp are labelled by azidopine (Bruggemann *et al.*, 1989, 1992; Yoshimura *et al.*, 1989), one in each half of the protein. However, it is believed that these two regions form a single drug binding site (Bruggeman *et al.*, 1992). It has, however, been suggested on the basis of binding and competition studies, that P-gp may have distinct binding sites for vinca alkaloids and azidopine (Tamai and Safa, 1991). Verapamil is more efficacious at inhibiting binding to the vinca alkaloid site. However, the steroid hormone progesterone competes equally well with either vinca alkaloids or azidopine for binding to P-gp (Yang *et al.*, 1989). As tamoxifen competes with both vinblastine and azidopine for binding to P-gp, if P-gp is able to bind or handle vinca alkaloids and azidopine differentially, then tamoxifen (like progesterone) must interfere with both sites/mechanisms.

Compounds which reverse multidrug resistance may prevent drug transport simply by occupying the drug binding site on P-gp, or by competing directly with cytotoxic drugs for transport. Many MDR reversing agents, such as verapamil (Cano-Gauci and Riordan, 1987; Yusa and Tsuro, 1989), azidopine (Tamai and Safa, 1991), cyclosporin and FK506 (Ueda *et al.*, 1992), have been reported to be transported by P-gp. However, we could detect no difference in the abilities of drug-resistant and drug-sensitive cells to accumulate tamoxifen. This suggests that tamoxifen is not transported by P-gp. A previous report has also suggested that tamoxifen is not a substrate for ATP-sensitive drug efflux in adriamycin-resistant P388 murine leukaemic cells (Kessel, 1986). The interaction of tamoxifen with P-gp may be similar to that of the steroid hormone progesterone. Like tamoxifen, progesterone inhibits azidopine photolabelling of P-gp (Yang *et al.*, 1989) and vinca alkaloid binding to P-gp-containing plasma membranes (Yang *et al.*, 1990) and increases the cellular levels of vinblastine (Yang *et al.*, 1989), yet does not appear to be a substrate for transport by P-gp (Yang *et al.*, 1989; Saeki *et al.*, 1993). Thus, there appear to be two classes of reversers of P-gp-mediated drug resistance. The first class consists of compounds which compete with cytotoxic drugs for binding and transport by P-gp. The second class of reversers, which includes tamoxifen and progesterone, compete for drug binding, thereby blocking transport of chemotherapeutic agents, but are not themselves transported.

In addition to its role in drug resistance, expression of P-gp is also associated with a cell volume-regulated chloride channel (Gill *et al.*, 1992; Valverde *et al.*, 1992). Tamoxifen is also a high-affinity inhibitor of this chloride channel, although it is not known whether or not this inhibitory effect is mediated through the interaction of tamoxifen with P-gp (Zhang *et al.*, 1994). The blockage of chloride channels by tamoxifen in the lens of the eye leads to opacity, suggesting a molecular mechanism by which tamoxifen might lead to visual impairment (Zhang *et al.*, 1994). Together with the present finding that tamoxifen binds to P-gp, these data suggest that tamoxifen may interact with a number of functionally important targets, with consequent implications for the therapeutic use of this anti-cancer drug.

**Abbreviations:** MDR, multidrug resistance; P-gp, P-glycoprotein; FBS, fetal bovine serum; DMEM, Dulbecco's modified Eagle medium; PMSF, phenylmethylsulfonyl fluoride; EDTA, ethylenediaminetetraacetic acid.

**Acknowledgements**
We thank Dr Michael M Gottesman for providing the cell lines used in this study. CFH is a Howard Hughes International Research Scholar. This work was supported by the Howard Hughes Medical Institute and the Imperial Cancer Research Fund.

**References**

BERTHOIS Y, KATZENELLENBOGEN JA AND KATZENELLENBOGEN BS. (1986). Phenol red in tissue culture media is a weak estrogen: implications concerning the study of estrogen-responsive cells in culture. *Proc. Natl Acad. Sci. USA*, **83**, 2496–2500.

BRUGGEMANN EP, GERMANN UA, GOTTESMAN MM AND PASTAN I. (1989). Two different regions of phosphoglycoprotein are photoaffinity labelled by azidopine. *J. Biol. Chem.*, **264**, 15483–15488.

BRUGGEMANN EP, CURRIER SJ, GOTTESMANN MM AND PASTAN I. (1992). Characterisation of the azidopine and vinblastine binding site of P-glycoprotein. *J. Biol. Chem.*, **267**, 21020–21026.

CALLAGHAN R AND RIORDAN JR. (1993). Synthetic and natural opiates interact with P-glycoprotein in multidrug resistant cells. *J. Biol. Chem.*, **268**, 16059–16064.

CANO-GAUCI DF AND RIORDAN JR. (1987). Action of calcium antagonists on multidrug resistant cells. *Biochem. Pharmacol.*, **36**, 2115–2123.

CHATTERJEE M AND HARRIS AL. (1990). Reversal of acquired resistance to adriamycin in CHO cells by tamoxifen and 4-hydroxytamoxifen: role of drug interaction with alpha 1 acid glycoprotein. *Br. J. Cancer*, **62**, 712–717.

CORNWELL MM, GOTTESMAN MM AND PASTAN I. (1986*a*). Increased vinblastine binding to membrane vesicles from multidrug resistant KB cells. *J. Biol. Chem.*, **261**, 7921–7928.

CORNWELL MM, SAFA AR, FELSTED RL, GOTTESMAN MM AND PASTAN I. (1986*b*). Membrane vesicles from multidrug resistant human cancer cells contain a specific 150- to 170-kDa protein detected by photoaffinity labelling. *Proc. Natl Acad. Sci. USA*, **261**, 7762–7770.

CORNWELL MM, PASTAN I AND GOTTESMAN MM. (1987). Certain calcium channel blockers bind specifically to multidrug resistant Human KB carcinoma membrane vesicles and inhibit drug binding to P-glycoprotein. *J. Biol. Chem.*, **262**, 2166–2170.

DEGREGORIO MW, FORD JM, BENZ CC AND WIEBE VJ. (1989). Toremifene: pharmacologic and pharmacokinetic basis of reversing multidrug resistance. *J. Clin. Oncol.*, **7**, 1359–1364.

GILL DR, HYDE SC, HIGGINS CF, VALVERDE MA, MINTENIG GM AND SEPULVEDA FV. (1992). Separation of drug transport and chloride channel functions of the human multidrug resistance P-glycoprotein. *Cell*, **71**, 23–32.

GOTTESMAN MM AND PASTAN I. (1993). Biochemistry of multidrug resistance by the multidrug transporter. *Annu. Rev. Biochem.*, **62**, 385–427.

HORIO M, GOTTESMAN MM AND PASTAN I. (1988). ATP-dependant transport of vinblastine in vesicles from human multidrug resistant cells. *Proc. Natl Acad. Sci. USA*, **85**, 3580–3584.

INABA M, KOBAYASHI H, SAKURAI Y AND JOHNSON RK. (1979). Active efflux of daunorubicin in sensitive and resistant sublines of P388 leukemia cells. *Cancer Res.*, **39**, 2200–2203.

JORDAN VC. (1990). In *Cancer Prevention*, DeVita Jr VT, Hellman S and Rosenberg SA (eds) pp. 1–12. J.B. Lippincott: Philadelphia.

JORDAN VC. (1992). Overview from the international conference on long term tamoxifen therapy for breast cancer. *J. Natl Cancer Inst.*, **84**, 231–234.

JORDAN VC AND NAYLOR KE. (1979). Binding of [$^3$H]oestradiol in the immature rat uterus during the sequential administration of anti-oestrogens. *Br. J. Pharmacol.*, **65**, 167–173.

JORDAN VC AND PRESTWICH G. (1977). Binding of [$^3$H]tamoxifen in rat uterine cytosols. A comparison of swinging bucket and vertical tube rotor sucrose density gradient analysis. *Mol. Cell Endocrinol.*, **8**, 179–188.

KARTNER N, RIORDAN JR AND LING V. (1983). Cell surface P-glycoprotein associated with multidrug resistance in mammalian cell lines. *Science*, **221**, 1285–1288.

KATZENELLENBOGEN JA, CARLSON KE, HEIMAN DF, ROBERTSON DW, WEI LL AND KATZENELLENBOGEN BS. (1983). Efficient and highly selective covalent labelling of the estrogen receptor with [$^3$H]tamoxifen aziridine. *J. Biol. Chem.*, **258**, 3487–3495.

KESSEL D. (1986). Interactions among membrane transport systems: anthracyclines, calcium antagonists and anti-estrogens. *Biochem. Pharmacol.*, **35**, 2825–2826.

KIRK J, HOULBROOK S, STUART NSA, STRATFORD IJ, HARRIS AL AND CARMICHAEL J. (1993*a*). Selective reversal of vinblastine resistance in multidrug resistant cell lines by tamoxifen, toremifene and their metabolites. *Eur. J. Cancer*, **29A**, 1152–1157.

KIRK J, HOULBROOK S, STUART NSA, STRATFORD IJ, HARRIS AL AND CARMICHAEL J. (1993*b*). Differential modulation of doxorubicin toxicity to multidrug and intrinsically drug resistant cell lines by anti-oestrogens and their major metabolites. *Br. J. Cancer*, **67**, 1189–1195.

LEONESSA F, JACOBSEN M, BOYLE B, LIPPMAN J, McGARVEY M AND CLARKE R. (1994). Effect of tamoxifen on the multidrug-resistant phenotype in human breast cancer cells: isobologram, drug accumulation and Mr 170,000 glycoprotein (gp170) binding studies. *Cancer Res.*, **54**, 441–447.

LERNER LJ AND JORDAN VC. (1990). Development of antiestrogens and their use in breast cancer: Eighth Cain Memorial Award Lecture. *Cancer Res.*, **50**, 4177–4189.

PETERS K AND RICHARDS FM. (1977). Chemical cross-linking: reagents and problems in studies of membrane structure. *Annu. Rev. Biochem.*, **46**, 523–551.

RAMU A, GLAUBIGER D AND FUKS Z. (1984). Reversal of acquired resistance to daunorubicin in P388 murine leukemia cells by tamoxifen and other triparanol analogues. *Cancer Res.*, **44**, 4392–4395.

RIORDAN JR AND LING V. (1985). Genetic and biochemical characterisation of multidrug resistance. *Pharmacol. Ther.*, **28**, 51-75.

RYFFEL B, WOERLY G, RODRIGUEZ C AND FOXWELL BMJ. (1991). Identification of the multidrug resistance-related membrane glycoprotein as an acceptor for cyclosporin. *J. Rec. Res.*, **11**, 675-686.

SAEKI T, UEDA K, TANIGAWARA Y, HORI R AND KOMANO T. (1993). Human P-glycoprotein transports cyclosporin A and FK506. *J. Biol. Chem.*, **268**, 6077-6080.

SAFA AR, GLOVER CJ, SEWELL JL, MEYERS MB, BIEDLER JL AND FELSTED RL. (1987). Identification of the multidrug resistance-related membrane glycoprotein as an acceptor for calcium channel blockers. *J. Biol. Chem.*, **262**, 7844-7888.

SHEN D, CARDARELLI C, HWANG J, CORNWELL M, RICHERT N, ISHII S, PASTAN I AND GOTTESMAN M. (1986). Multiple drug resistant human KB carcinoma cells independently selected for high level resistance to colchicine, adriamycin or vinblastine show changes in expression of specific proteins. *J. Biol. Chem.*, **261**, 7762-7770.

STUART NSA, PHILLIP P, HARRIS AL, TONKIN K, HOULBROOK S, KIRK J, LIEN EA AND CARMICHAEL J. (1992). High-dose tamoxifen as an enhancer of etopside cytotoxicity. Clinical effects and *in vitro* assessment in P-glycoprotein expressing cells. *Br. J. Cancer*, **66**, 833-839.

TAMAI I AND SAFA AR. (1991). Azidopine noncompetitively interacts with vinblastine and cyclosporin A binding to P-glycoprotein in multidrug resistant cells. *J. Biol. Chem.*, **266**, 16796-16800.

UEDA K, OKAMURA N, HIRAI M, TANIGAWARA Y, SAEKI T, KIOKA N, KOMANO T AND HORI R. (1992). Human P-glycoprotein transports cortisol, aldosterone and dexamethasone but not progesterone. *J. Biol. Chem.*, **267**, 24248-24252.

VALVERDE MA, DIAZ M, SEPULVEDA FV, GILL DR, HYDE SC AND HIGGINS CF. (1992). Volume regulated chloride channels associated with the human multidrug resistance P-glycoprotein. *Nature*, **355**, 830-833.

WIGLER PW AND PATTERSON FK. (1993). Inhibition of the multidrug resistance efflux pump. *Biochim. Biophys. Actua*, **1154**, 173-181.

YANG C-PH, DE PHINO SG, GREENBERGER LM, HSU SI-H AND HORWITZ SB. (1989). Progesterone interacts with P-glycoprotein in multidrug resistant cells and in endometrium of gravid uterus. *J. Biol. Chem.*, **264**, 782-788.

YANG C-PH, COHEN D, GREENBERGER LM, HSU SI-H AND HORWITZ SB. (1990). Differential transport properties of two mdr gene products are distinguished by progesterone. *J. Biol. Chem.*, **265**, 10282-10288.

YOSHIMURA A, KUWAZURI Y, SUMIZAWA T, ICHIKAWA M, IDEDA S, UDA T AND AKIYAMA S. (1989). Cytoplasmic orientation and two-domain structure of the multidrug transporter, P-glycoprotein, demonstrated with sequence specific antibodies. *J. Biol. Chem.*, **264**, 16282-16291.

YUSA K AND TSURO T. (1989). Reversal mechanism of multidrug resistance by verapamil: direct binding of verapamil to P-glycoprotein on specific sites and transport of verapamil outward across the plasma membrane of K562 ADM cells. *Cancer Res.*, **49**, 5002-5006.

ZHANG JJ, JACOB TJC, VALVERDE MA, HARDY SP, MINTENIG GM, SEPULVEDA FV, GILL DR, HYDE SC, TREZISE AEO AND HIGGINS CF. (1994). Tamoxifen blocks chloride channels: a possible mechanism for cataract formation. *J. Clin. Invest.*, **94**, 1690-1697.